# Exploring the causality and pathogenesis of systemic lupus erythematosus in breast cancer based on Mendelian randomization and transcriptome data analyses

**DOI:** 10.3389/fimmu.2022.1029884

**Published:** 2023-01-16

**Authors:** Wenjie Li, Rong Wang, Wei Wang

**Affiliations:** Department of Radiation Oncology, Nanfang Hospital, Southern Medical University, Guangzhou, China

**Keywords:** systemic lupus erythematosus, breast cancer, Mendelian randomization, transcriptomic data, prevention

## Abstract

**Introduction:**

There has been a cumulative interest in relationships between systemic lupus erythematosus (SLE) and cancer risk. Breast cancer is the most common cancer among women worldwide. However, the casual association and pathogenesis between SLE and breast cancer remains incompletely unknown.

**Methods:**

Mendelian randomization (MR) analysis was first conducted to investigate the potential causality between SLE and breast cancer. Sensitivity analyses were applied to validate the reliability of MR results. Transcriptomic data analyses based on the Cancer Genome Atlas and Gene Expression Omnibus databases were then performed to identify and construct a SLE-related gene signature (SLEscore).

**Results:**

The MR analysis demonstrated that genetic predisposition to SLE was casually associated with the decreased risk of breast cancer in the East Asian cohort (odds ratios: 0.95, 95% confidence interval: 0.92-0.98, *p*=0.006). However, no casual associations were observed in the European population. Furthermore, sensitivity analyses proved the robustness of the present MR results. A prognostic SLEscore consisting of five SLE-related genes (*RACGAP1, HMMR, TTK, TOP2A*, and *KIF15*) could distribute patients with breast cancer into the high- and low-risk groups according to survival rates with good predictive ability (*p* < 0.05).

**Conclusion:**

Our MR study provided evidence that genetic changes in SLE were significantly associated with the decreased risk of breast cancer in the East Asian population, while no causality was found in the European cohorts. Transcriptome data analyses indicated that the SLEscore could serve as a novel biomarker for predicting prognosis when breast cancer and SLE coexisted in patients.

## Introduction

Interest in the duality of the immune system in cancer has rumbled on for years because inflammation can accelerate tumorigenesis, whereas the immune system possesses powerful anti-tumor properties once activated. Systemic lupus erythematosus (SLE), an intricately and systematically chronic inflammation, is typically an autoimmune disease (1). SLE patients were reported to have at most five-times the risk of mortality from cancers compared with the general population (2–4). Consequently, more attention should be paid to the risks of tumorigenesis in SLE patients. Breast cancer is the most common cancer among women worldwide and strongly linked to chronic inflammation (5, 6). Due to the high incidence of SLE in females, accumulating observational or cohort studies (7–9) have explored the association between SLE and breast cancer. However, observational studies yielded conflicting conclusions because results may be influenced by many potential confounding factors, including sample size and anti-SLE immunosuppressive therapy (10). Epidemiologic patterns of SLE and breast cancer might also vary between different ethnic populations (11). Thus, the causal relationship between SLE and breast cancer risk needs to be assessed with a more well-designed approach.

Regarded as a promising epidemiological method, the Mendelian randomization (MR) analysis was proposed for precise assessment of potential causality between exposures and outcomes (12). MR has been likened to randomized controlled trials where a random assortment of alleles contributes to a random assignment of exposures (12). Moreover, the MR method is independent on environmental risk factors and prior to disease progression. Therefore, in order to avoid reverse causality and potential confounding factors, genetic variants are utilized as the instrumental variables (IVs) in the MR analysis. Using summary-level statistics from previous genome-wide association studies (GWASs) and transcriptomic data, we aimed to perform a MR analysis to more feasibly explore the possible causality between SLE and breast cancer. Besides, SLE would increase mortality in patients with breast cancer when both pathologies coexist (11). Thus, another purpose of this study was to investigate common molecular mechanisms between two diseases by using differentially expressed genes (DEGs) for risk stratification and therapeutic targets.

## Method

**Figure 1:**
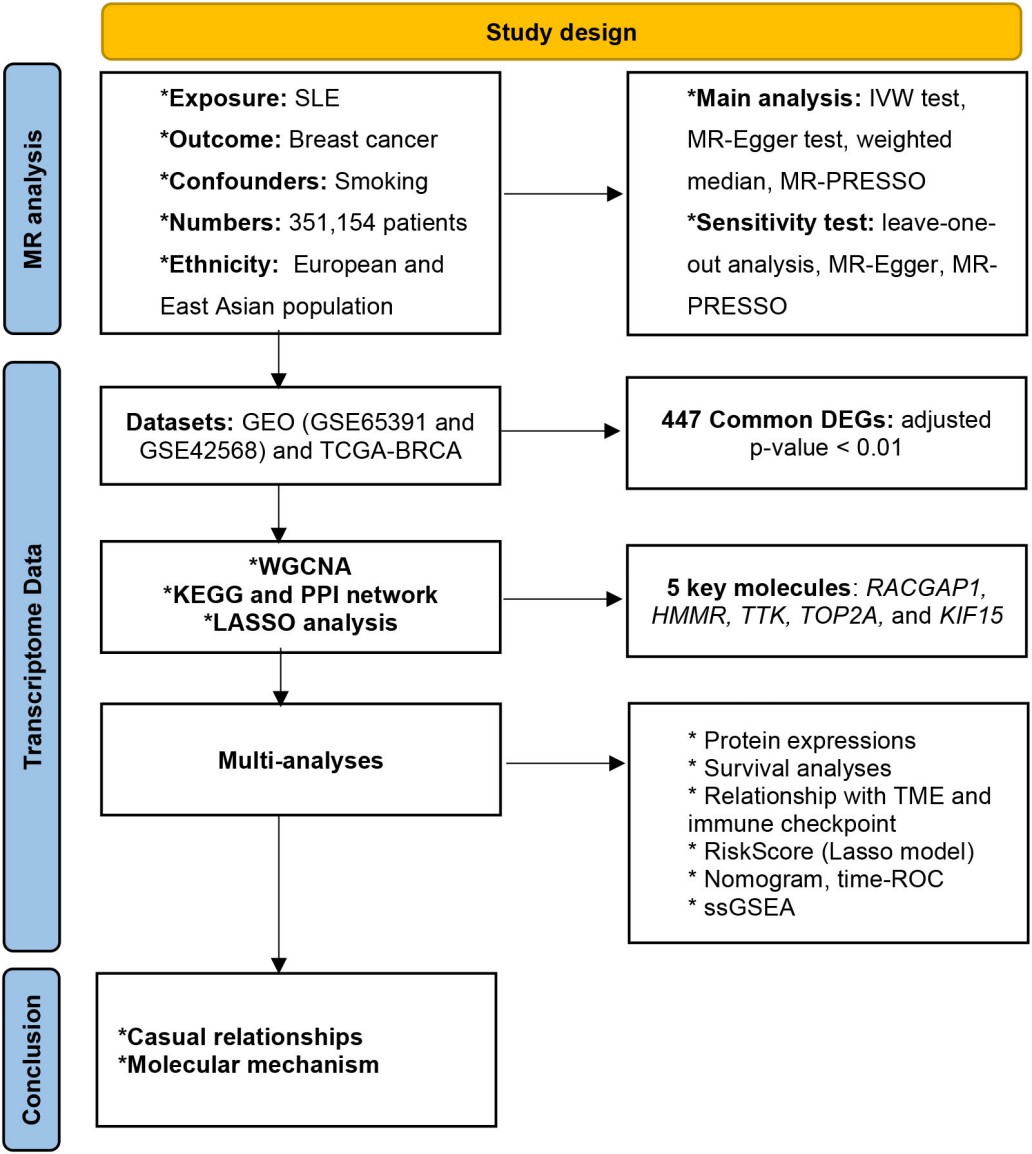
Overview of study design.

The overview design of our work was shown in **Figure 1**. Data sources for MR and transcriptome data are publicly available online.

## Mendelian randomization analysis

### Data sources

Our work was conducted based on summary-level data from GWASs of European and East Asian ancestries, respectively (**Table 1**).

- SLE Effect estimates of the SNPs associated with SLE risk were acquired from a study comprising 5,201 cases and 9,066 controls of European ancestry (13). The SLE GWAS of Wang et al. (14), which increased the sample size of East Asian populations to the level of existing European studies and enrolled 12,653 people, was also utilized.
- Breast cancer Genetic instruments in breast cancer were extracted from a large GWAS study with 122,977 cases and 105,974 controls of European ancestry (15). Variants of East Asian population involving 5,552 patients and 89,731 controls were obtained from the Japanese biobank (GWAS trait ID: bbj-a-160).

**Table 1:** Details of GWASs analyzed in the present MR analyses.

Phenotype	First author or Consortium	Sample size	Number of patients	Number of controls	Number of variants	Ethnicity	F-statistic	Pubmed ID	Trait ID in GWAS	Year
Exposure										
Systemic lupus erythematosus	Bentham J	14267	5201	9066	7071163	European	Not Applicable	26502338	ebi-a-GCST003156	2015
Systemic lupus erythematosus	Wang YF	12653	4222	8431	5691661	East Asian	Not Applicable	33536424	ebi-a-GCST90011866	2021
Outcome										
Breast cancer	BCAC	228951	122977	105974	10680257	European	11279	29059683	ieu-a-1126	2017
ER+ Breast cancer	BCAC	175475	69501	105974	10680257	European	11279	29059683	ieu-a-1127	2017
ER- Breast cancer	BCAC	127442	21468	105974	10680257	European	11279	29059683	ieu-a-1128	2017
Breast cancer	Japanese Biobank	95283	5552	89731	8872152	East Asian	128.31	NA	bbj-a-160	2020

MR, Mendelian Randomization; GWAS, genome-wide association studies; BCAC, Breast Cancer Association Consortium; NA, not available.

### Generation of genetic instruments

All MR approaches were based on three core assumptions to minimize the influence of bias on the MR estimates (16): (i) the IVs have a strong relationship with SLE, (ii) the IVs influence breast cancer only through their effects on SLE, and (iii) the IVs are independent of any other confounding parameters. Violations of three core assumptions would cause unreliable conclusions (**Figure S1**). Hence, the following steps would help to choose the best IVs associated with SLE.

First, single-nucleotide polymorphisms (SNPs) robustly correlated with SLE were collected from the published GWAS at a threshold of statistical significance (p<5×10^−8^). Second, we conducted an exclusion if mutual linkage disequilibrium (LD) shared the larger p-value conjugately and exceeded the limited value (window size = 10,000 kb, R^2^ < 0.001) by means of LD analysis. Third, the F-statistic was assessed using the formula $F=\frac{R2\left(N-k-1\right)}{k\left(1-R2\right)}$ to evaluate potential instrument bias. Herein, N, k, and R^2^ denoted sample size, the number of SNPs, and the proportion of variance explained by the IVs, respectively. If the F-statistic < 10, the value of IVs used in the present study was weak.

### Two-sample and multivariate MR analysis

An inverse variance-weighted (IVW) meta-analysis of the Wald ratio estimates was performed to explore the causal effect of SLE on the risk of breast cancer. Because the IVW test presents a weighted regression of outcomes on exposures with the intercept constrained to zero, the estimate might be biased. In this case, more sensitive methods, including the MR-Egger regression (17) and weighted-median test (18) were conducted.

The MR-Egger model could assess the causal effect from the weighted regression of the IVs-breast cancer relationships on the IVs-SLE associations, and the intercept presented the average pleiotropic effect. The casual estimates would be provided by the weighted median analysis if over 50% IVs are effective. Moreover, basic MR analyses could not provide an accurate estimate of the causality of SLE on the risk of breast cancer if we included SLE-associated genetic variants that were also related to confounders. Smoking is one of the most well-known risk factors causing breast cancer (19). Moreover, smoking can trigger SLE in genetically-predisposed individuals (20). Hence, smoking was a potential confounder of the RA-breast cancer correlation. To estimate the independent impact of the traits associated with SLE, the multivariable MR method was applied to adjust the effects from the potential confounder (Smoking-Trait ID: ieu-b-4877).

### Removal of horizontal pleiotropy and sensitivity analyses

The Mendelian Randomization Pleiotropy Residual Sum and Outlier (MR-PRESSO) analysis (21), leave-one-out analyses and Cochran’s Q test were conducted to extensively assess the MR results. Of note, the MR-PRESSO method can correct the presence of horizontal pleiotropy by removing outlying SNPs (21). Leave-one-out analysis could analyze the influence of outlying values. Cochran’s Q test identified SNPs that were responsible for heterogeneity based on the IVW and the MR-Egger estimates (22).

## Transcriptomic analyses

### Data acquisition

The microarray expression and clinical data of GSE65391 (924 SLE and 48 controls) was downloaded from the Gene Expression Omnibus (GEO) database. The RNA-sequencing data with corresponding clinical information of 130 normal tissues and 1208 breast cancer samples were acquired from the Cancer Genome Atlas (TCGA) and GSE42568.

### Identification of DEGs

447 overlapping SLE- and breast cancer- DEGs were identified by the “limma” R package (23) with the threshold |log_2_FC| >1 and adjusted p-value < 0.01. Subsequently, 447 DEGs were subjected to construction of co-expression networks using Weighted Gene Co-Expression Network Analysis (WGCNA) (24).

### Functional enrichment analysis and protein–protein interaction

To further investigate the biological mechanisms of DEGs, Gene Ontology (GO) analysis and Kyoto Encyclopedia of Genes and Genomes (KEGG) annotation were conducted using the “ClusterProfiler” R package (25). The PPI network was conducted to find out the top twenty pivotal module genes using STRING database (https://string-db.org). Spearman correlation analyses were performed to evaluate the existence of the correlations among the top twenty genes.

### Development and validation of the SLE-related prognostic model

First, the intersection genes associated with overall survival (OS) were calculated by least absolute shrinkage and selection operator (LASSO) regression using the ‘glmnet’ R package. LASSO algorithm was herein applied to solve redundancy problem caused by collinearity among SLE-related DEGs in the TCGA-BRAD database. A best subset of DEGs related to breast cancer prognosis was identified by shrinkage of the regression coefficient. A SLE-related prognostic model (SLEscore) of five genes (namely *RACGAP1, HMMR, TTK, TOP2A*, and *KIF15*) was constructed. The SLEscore was defined as follows: $\sum_{x=1}^{n}coefficient\;x*expression\;x$ . The TCGA cohort with breast cancer from were distributed into low- and high-risk groups based on the best cut-off risk SLEscore, and OS would be compared between the two subgroups with Kaplan-Meier analysis. Multivariable Cox hazards models with a concordance index (C-index) were utilized to analyze the discrimination of clinico-SLEscore variables. Time receiver-operating characteristic (ROC) analysis with area under curve (AUC) was used to evaluate the prognostic performance of the SLEscore. Besides, GSE42568 containing 104 BRAC and 17 normal samples was applied as the validation set to verify the predictive value of SLEscore. The SLEscore for the GSE42568 cohort was estimated using the same formula obtained from the TCGA cohort, and patients were divided into SLEscore^low^ and SLEscore^high^ subgroups based on the best cut-off value.

### Nomogram based on the SLEscore

A predicted nomogram based on the results of multivariate Cox analysis was developed by the “rms” R package to assess the prediction of 1-, 3-, and 5-year OS probability. Calibration curves could estimate the consistency between nomogram-predicted probabilities and noted probabilities.

### Estimation of immune infiltration cells and ESTIMATEscore

The enrichment scores calculated by the single-sample gene-set enrichment analysis (ssGSEA) (26) were analyzed to demonstrate the abundance of 24 human tumor immune environment (TME) infiltration cells. The ESTIMATE algorithm was performed to calculate the stromal score and immune score that can forecast the purity of a tumor according to the infiltration of stromal cells and immunocytes (27).

### Evaluation of immune checkpoint blockade and PANoptosis

PANoptosis is an inflammatory programmed cell death which is triggered by the contemporaneous engagement of components from pyroptosis, apoptosis, and/or necroptosis (28). Pearson coefficient was calculated to estimate correlations between the SLEscore and gene markers of ICB, apoptosis (29), pyroptosis (30, 31) and necroptosis (32).

### Statistical analysis

R software (Version 4.0.3) was applied to conduct all the data analyses in the present study. The present MR study was performed in accord with the recommended items (supplemental file: Checklist item). All MR analyses were carried out by the “Two-Sample MR” and “MRPRESSO” packages. Odds ratios (ORs), hazard ratios (HRs) with corresponding 95% confidence intervals (CIs) were calculated. Two-sided p-values less than 0.05 were considered to be statistically significant.

## Result

### Selection of SNPs

In general, this MR study analyzed a total of 243,218 European-descent individuals (128,178 cases and 115,040 controls) and 107,936 people of East Asian descent (9,774 cases and 98,162 controls). We extracted IVs which were significantly associated with SLE from the GWAS (*p*< 5 × 10^−8^) and removed LD (r^2^<0.001,10,000-kb). Besides, the F-statistic in our analysis were greater than 100 (**Table 1**), indicating that the IVs powerfully predict the incidences of SLE.

### Genetic susceptibility to SLE and breast cancer risk

**Figure 2:**
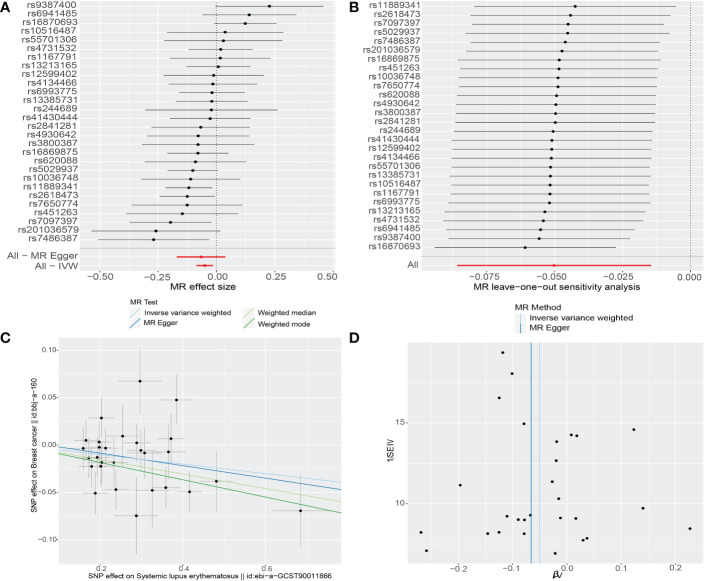
The causality of systemic lupus erythematosus on breast cancer risk in East Asians. **(A)** Forest plot. The red points demonstrate the integrated estimates using all SNPs together, using IVW method. Horizontal lines represent 95% confidence intervals. **(B)** Leave-one-out analysis. Black points depict the IVW method was used to assess the causal effect, excluding single specific variant from the analysis. The red point denotes the inverse-variance weighted estimate using all SNPs. **(C)** Scatter plot. The slope of each line denotes the estimated effect of per mendelian randomization method. **(D)** Funnel plot. Vertical lines represent estimates with all SNPs. Symmetry in the funnel plot demonstrates no obvious horizontal pleiotropy. IVW, Inverse‐variance weighted; SNP, single-nucleotide polymorphism.

As shown in **Table 2**, MR analyses revealed null causal associations between SLE and breast cancer in the European cohort (breast cancer: OR 0.9985, 95%CI 0.9873-1.0099, *p*=0.79; ER+ breast cancer: OR 0.9974, 95% CI 0.9850-1.0101, *p*=0.69; ER- breast cancer: OR 1.009, 95% CI 0.99-1.02, *p*=0.22). There was no evidence indicating that an increased risk of breast cancer based on the other MR methods (**Table 2**). However, the casual inference of genetic liability to SLE and breast cancer in East Asian population was noted (IVW: OR: 0.95, 95%CI: 0.92-0.98, *p*=0.006; weighted median: OR: 0.93, 95%CI: 0.88-0.97, *p*=0.002; MR-PRESSO: OR 0.95, 95%CI: 0.92-0.98, *p*=0.004) (**Table 2**, **Table S2** and **Figure 2**). Multivariate MR analysis also supported the finding that SLE was significantly associated with breast cancer in the East Asian population (SNPs: 25, OR: 0.95, 95%CI: 0.92-0.98, *p*=0.0013) after adjusting the confounder (smoking, trait-ID: ieu-b-4877).

**Table 2:** Mendelian randomization estimates of the casual relationships between SLE and breast cancer risks.

Exposure	PubmedID	Ethnicity	nSNPs	IVW method		Weighted median method		MR-Egger	
				OR (95% CI)	p value	OR (95% CI)	p value	OR (95% CI)	p value
Breast cancer	29059683	European	39	0.9985 (0.9873-1.0099)	0.79	0.9997 (0.9878-1.0119)	0.96	0.99 (0.97-1.02)	0.52
ER+ Breast cancer	29059683	European	39	0.9974 (0.9850-1.0101)	0.69	0.9999 (0.9856-1.0143)	0.98	0.99 (0.96-1.02)	0.56
ER- Breast cancer	29059683	European	39	1.009 (0.99-1.02)	0.22	1.01 (0.99-1.03)	0.25	1.01 (0.98-1.04)	0.35
Breast cancer	NA	East Asian	28	0.95 (0.92-0.98)	0.006*	0.93 (0.88-0.97)	0.002*	0.94 (0.84-1.04)	0.23

SLE, systemic lupus erythematosus; IVW, inverse-variance weighted; MR, mendelian randomization; OR, odds ratio; CI, confidence interval; NA, not available; *, statistically significant. The bold values means statistical significance.

### Sensitivity analyses for MR estimates

First, we performed MR-Egger regression to investigate horizontal pleiotropy, and the results confirmed that pleiotropy was unlikely to bias the causal relationship (all *p*-values > 0.05) (**Table S1**). Second, the results of MR-PRESSO tests were in line with ones of IVW methods without outliers, which suggested that the original results are reliable (**Table S2**). Third, given the potential relationship between SLE and breast cancer in the East Asian cohort, we conducted leave-one-out analyses and Cochrane Q tests. The leave-one-out analysis discovered no single SNP which drove the causal link between SLE and breast cancer (**Figure 2**). The *p*-values of Cochrane Q tests were all greater than 0.05 (Q value for the IVW test: 32.93, *p*=0.2; Q value for the MR-Egger test: 32.8, *p*=0.17), indicating no heterogeneity between SNPs.

### Identification of SLE−related DEGs in patients with breast cancer

**Figure 3:**
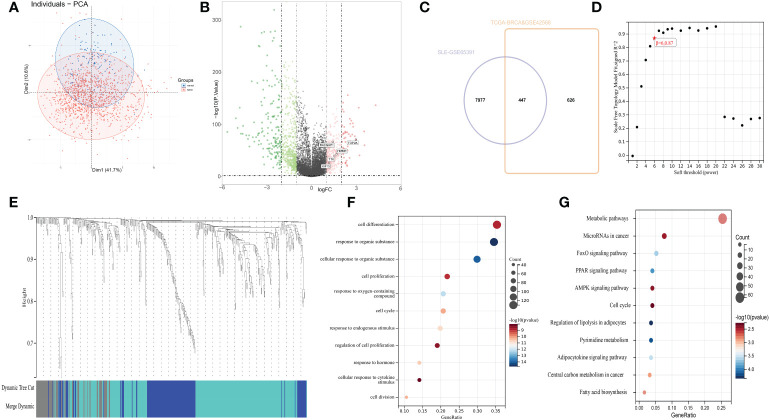
**(A)** Principal component analysis for tumor samples and normal breast tissues. **(B)** The volcano plot presents the expression pattern of DEGs in the coalescent cohort of TCGA and GSE42568. Green, downregulated genes. Red, up-regulated genes. **(C)** Venn diagram showing the intersecting 447 SLE-related differentially genes associated with overall survival. |log_2_FC| >1, adjusted p-value < 0.01. **(D)** Analysis of scale-free index for the different soft-thresholding powers. the appropriate soft-power was 6. **(E)** Clustering dendrogram of 447 genes according to the measurement of dissimilarity. Genes are hierarchically divided into three modules with different colors. **(F)** Gene ontology enrichment analysis. **(G)** Kyoto Encyclopedia of Genes and Genomes enrichment analysis.

After standardizing the microarray results (**Figures 3A, B**), 447 common DEGs between SLE- and breast cancer-related datasets were recognized (**Figure 3C**). WGCNA (soft threshold power=6) further removed 61 obvious outliers in the grey module by clustering, and recognize 386 hub genes of interest (**Figures 3D, E** and **Table S3**).

### Analysis of the functional characteristics

To further figure out the latent functions of 386 SLE-DEGs in breast cancer, we conducted GO and KEGG enrichment analyses. GO analysis showed DEGs were enriched in cell cycle, cell proliferation, and response to hormone (**Figure 3F**). KEGG enrichment analysis were mainly involved in cancer- and cell cycle-related pathways including metabolic pathways, microRNAs, transcriptional mis-regulation, proteoglycans, and central carbon metabolism (**Figure 3G**).

### PPI network and analysis of hub genes

**Figure 4:**
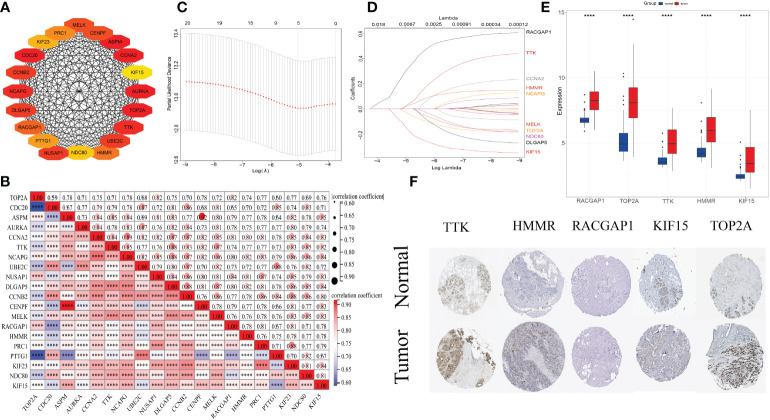
**(A)** Protein–protein interaction network of top 20 hub clustering genes. **(B)** Spearman correlations among 20 genes. The size of circle denotes the correlation intensity. ****, p<0.0001. **(C, D)** The least absolute shrinkage and selection operator regression model to evade overfitting of recurrence features and narrow the range of SLE-related key differentially expressed genes. **(E)** Expression levels of 5 prognostic genes in the normal tissues and cancer samples. **(F)** Immunohistochemical results revealed that expression levels of 5 prognostic molecules were higher in tumor samples than those in normal breast tissues.

First, the PPI network of were constructed for the 386 common DEGs. Second, Cytuhubba plug-in of Cytoscape was used to calculated top 20 hub genes (*AURKA, UBE2C, CDC20, PTTG1, CCNB2, MELK, NDC80, CENPF, PRC1, KIF23, TOP2A, RACGAP1, NUSAP1, HMMR, ASPM, KIF15, TTK, DLGAP5, CCNA2*, and *NCAPG*) (**Figure 4A**). Third, spearman correlation analysis exhibited remarkably close connections among twenty hub genes (all *p*-values<0.0001) (**Figure 4B**).

### Construction and verification of the prognostic model

**Figure 5:**
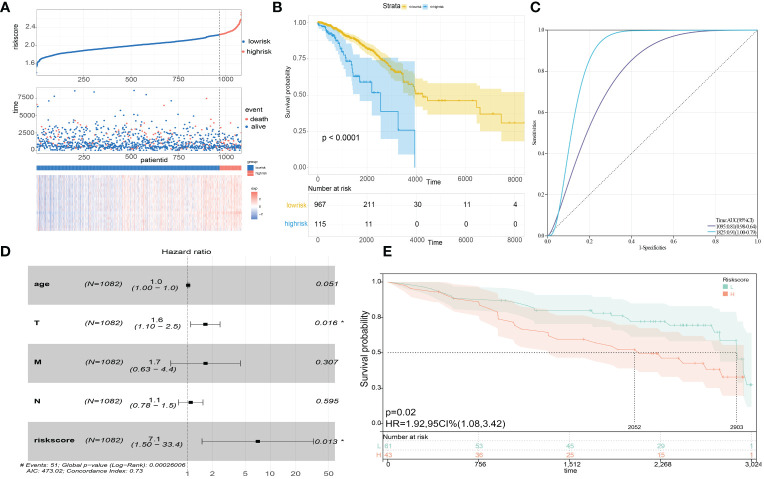
**(A)** Risk score distribution, survival status scatter plots, and gene expression heatmap in the prognostic model. **(B)** Kaplan-Meier survival analysis of the high- and low-risk SLEscore groups. Blue, the high-risk group. Yellow, the low-risk group. **(C)** The time receiver-operating characteristic curve showing the accuracy of the SLEscore. 3-year AUC: 0.81, 5-year AUC: 0.91. **(D)** Multivariate COX regression analysis indicating the riskscore signature was an independent risk factor. **(E)** The Kaplan-Meier curve of overall survival in the GSE42568 cohort to validate the predictive power of the SLEscore. Blue, the low-risk group. Red, the high-risk group.

**Figure 6:**
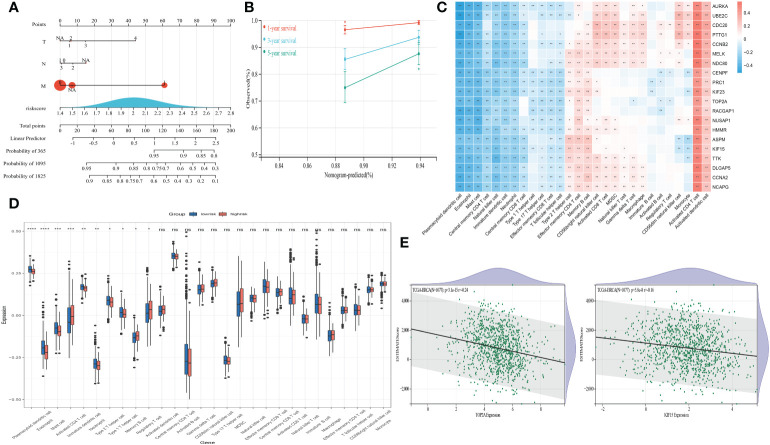
**(A)** Construction of a nomogram for overall survival prediction in patients with breast cancer. **(B)** Calibration plot of 1-, 3- and 5-years actual risk probability was exhibited, indicating moderate power for predicting survival for patients with breast cancer. **(C)** Spearman correlations presenting the correlation between 24 kinds of immune cells and prognostic genes. Red, positive correlation. Blue, negative correlation. **(D)** Differential expression of 24 kinds of immune cells in the high- and low-risk SLEscore groups. Blue, the low-risk group. Red, the high-risk group. ****, p<0.0001; ***, p<0.001; **, p<0.01; *, p<0.05; ns, no significance. **(E)** The correlations between the ESTIMATE scores and the expression levels of TOP2A and KIF15.

LASSO regression method was utilized to refine twenty hub genes. Finally, the most valuable five predictive genes (*RACGAP1, HMMR, TTK, TOP2A*, and *KIF15*) were selected for the construction of SLEscore (**Figures 4C, D**). The SLEscore was calculated as follows: [-0.036 × Expression value of *TOP2A*] + [0.032 × Expression value of *TTK*] + [0.32 × Expression value of *RACGAP1*] + [0.024 × Expression value of *HMMR*] + [-0.16 × Expression value of *KIF15*]. All five hub genes of the SLEscore were significantly up-regulated in tumor samples (**Figures 4E–G**). Patients with breast cancer were divided into two subgroups according to the SLEscore, in which the SLEscore^high^ was related to the higher expression levels of five prognostic molecules (**Figure 5A**). Compared with the low-SLEscore group, the high-SLEscore group was associated with noticeable worse OS (**Figure 5B**). The ROC curve indicated that SLEscore could be a sensitive marker for predicting OS of patients with breast cancer (3-year AUC: 0.81, 5-year AUC: 0.91) (**Figure 5C**). Furthermore, the multivariate COX regression analysis presented that the SLEscore was an independent risk factor of patients with breast cancer (HR 7.1, 95%CI 1.50-33.4, *p*=0.013) (**Figure 5D**). The C-index of the SLEscore we established was 0.73 (standard error: 0.043). The SLEscore was further corroborated in the GSE42568 dataset, suggesting that the SLEscore constructed using the TCGA database was an independent prognostic factor for patients with breast cancer (HR 1.92, 95%CI 1.08-3.42, *p*=0.02) (**Figure 5E**). Afterward, we built a nomogram for patients with breast cancer by integrating SLEscore, age, and TNM stage, which performed well in predicting the 1-, 3- and 5-year OS in patients with breast cancer (**Figures 6A, B**).

### Exploration of TME cells, ESTIMATEscore, ICB and PANoptosis

Spearman correlation analyses disclosed a significantly association between twenty molecules and TME infiltrating cells (**Figure 6C**). Notably, the high-SLEscore was significantly related to lower dendritic cell, eosinophil, mast cell, CD4+ T cell, and helper T cell expressions (**Figure 6D**). The ESTIMATEscores were negatively correlated with the expression levels of five hub genes (all *p*-values<0.05) (**Figure 6E** and **Figures S2**–**S4**), indicating the relationship to disease outcomes and tumor-infiltrating immune environment. The SLEscore was significantly associated with eight kinds of ICB and gene patterns of PANoptosis (**Table 3**), which were proved to be prognostic biomarkers in patients with breast cancer.

**Table 3:** Correlations of gene markers.

Type	Gene marker	*SLEscore*	
		Cor	P-value
ICB			
	*PD-L1*	0.14	9.1e-0.7*
	*Lag3*	0.19	1.2e-11*
	*CTLA-4*	0.2	1e-12*
	*CD47*	0.3	0*
	*TIM*	0.17	7.5e-09*
	*TIGIT*	0.18	1e-09*
	*HAVCR2*	0.17	7.5e-09*
	*DNAM-1*	0.15	2.8e-0.7*
Apoptosis			
	*TNF*	0.22	3.2e-14*
	*Bcl-2*	-0.076	0.0082*
	*SMAC*	0.38	0*
	*NLRP3*	-0.26	1.4e-19*
	*XIAP*	0.15	1.9e-07*
Pyroptosis			
	*SEMA3B*	-0.1	0.00053*
	*IGKC*	0.013	0.64
	*KLRB1*	-0.14	5.9e-07*
	*BIRC3*	0.082	0.0046*
	*PSME2*	0.29	0*
	*GZMA*	0.068	0.018*
	*GZMB*	0.19	2.4e-11*
	*IL18*	0.11	8.5e-05*
	*IRF1*	0.16	1.4e-08*
Necroptosis			
	*FASLG*	0.15	1.2e-07*
	*IPMK*	0.24	0*
	*FLT3*	0.06	0.0036*
	*SLC39A7*	0.24	0*
	*HSP90AA1*	0.43	0*
	*LEF1*	0.08	0.0079*

*, statistically significant; Cor, the value of Pearson’s correlation; ICB, immune checkpoint blockade. The bold values means statistical significance.

## Discussion

Immunity has continuously been a vital direction to study in the progression of cancers. However, no convincing evidence exists that SLE is related to the mechanisms of tumorigenesis. To avoid potential confounders and inverse causation, the present MR analysis was designed to evaluate the causality between SLE and breast cancer risk with less susceptibility. Our MR results demonstrated that genetical predisposition to SLE was associated with a decreased risk of breast cancer in East Asian-descent individuals. However, no causal associations of genetic liability to SLE with breast cancer were observed in the European populations. Furthermore, when two serious diseases coexist, a SLE‐related DEGs risk signature (SLEscore) could stratify patients with breast cancer into two groups and predict the clinical endpoints. The SLEscore was further validated to be remarkedly correlated with the levels of tumor-infiltrated immune cells, ESTIMATE scores, ICB, and PANoptosis.

Accumulating evidence indicated that SLE was related to an incidence of cancers affecting multiple organs as well. Previous studies has showed SLE may be an independent risk factor for developing lymphoma, pancreatic, cervical, thyroid, lung, ovarian, and oral cancers (3, 4). Nonetheless, other evidence revealed that SLE was associated with reductions of several malignancies (melanoma, prostate, endometrial, and uterine cancers) (33). As a result of high incidences of SLE and breast cancer in women, many efforts have been made to investigate the correlations between SLE and breast cancer. However, the most recent pooled data from observational or cohort studies failed to find an increased risk of breast cancer in SLE patients (2, 7, 34). These results were partly consistent with our findings that SLE was not a risk factor of breast cancer in Europeans. Compared with previous findings from observational study designs, our MR studies may provide more robust evidence. Several potential confounding factors, including time periods, environmental exposures, and population-specific genetics in observational studies might make results more or less affected. However, MR analysis can minimize the effect of confounders and serve as a potential mimic of randomized controlled trials. Moreover, racially diverse may lead to a different breast cancer risk in SLE patients (11). Previous research did not take the heterogeneity of the study populations into account. In the present study, we elucidated that genetically tendency to SLE was related to a decreased risk of breast cancer in East Asian-descent populations. The racial differences of breast cancer risk observed in SLE patients may indicate different breast cancer prevention strategies.

Although we found that females with SLE were not at increased risk for developing breast cancer, it is of paramount importance to assess the outcomes of the coexistence of two diseases, since current evidence suggested that SLE may be detrimental for breast cancer outcomes (11). Elderly breast cancer patients with SLE had worse 5- and 8-year OS when compared to those without SLE (11). Another cohort study validated that SLE patients with cancers had an increased risk of mortality (35). One of the probable explanations for excess mortality may be comorbidities, which were increased in patients with both SLE and breast cancer. Additionally, some of the differences in OS may be explained by both SLE and breast cancer therapies. For example, patients with both conditions were less likely to receive corticosteroids or antimalarials compared to those with SLE without breast cancer (11). Radiotherapy is an another important treatment for breast cancer, but radiotherapy may conceivably worsen SLE flares, and thus be withheld from SLE patients when compared to general patients (36). Moreover, a recent study indicated that SLE might increase mortality in elder women with breast cancer due to corticosteroid insufficiency.

Given the detrimental effects of SLE on breast cancer, we performed WGCNA to identify 386 SLE-related DEGs in 130 normal breast samples and 1208 breast cancer samples. We noticed that the genomic difference exhibited a significant correlation with cancer or cell cycle-related pathways, including metabolic pathways, transcriptional mis-regulation, and fatty acid biosynthesis. These findings were consistent with some earlier prior studies in which the presence of aberrant transcription (37) or fatty acid metabolic reprogramming (38) was ascertained to be a crucial driver of breast cancer progression. The PPI network further introduced the top twenty molecules, which exhibited substantially close interactions in breast cancer. The novel prognostic SLEscore with five key therapeutic targets (*RACGAP1, HMMR, TTK, TOP2A*, and *KIF15*) was developed for the management of breast cancer patients with SLE using the LASSO algorithm. Several experiments have shown the roles of these five genes in breast cancer. *RACGAP1* modulates mitochondrial quality control to drive breast cancer metastasis (39). The overexpression of *HMMR* increases breast cancer-mutant tumorigenesis by modifying the cancer cell phenotype and TME (40). *TTK* expression levels are associated with mesenchymal and proliferative phenotypes in breast cancer (41). A high *TOP2A* gene dosage has a strong inverse prognostic impact (42). *KIF15* promotes tumor proliferation and migration in breast cancer, thus resulting in a significantly worse prognosis (43). The present study also supported the above experimental findings, indicating that high expression levels of five pivotal molecules were observed in breast cancer samples, and low expression levels were related to prolonged OS.

We therefore constructed the SLEscore based on SLE-related DEGs as well as a nomogram to predict the prognosis of breast cancer patients complicated with SLE. Multivariable Cox models, KM survival analyses, and ROC curves further confirmed the predictive accuracy of the SLEscore. The latest research demonstrated that the addition of ICB to chemotherapy could contribute to significantly longer OS than chemotherapy alone (44). In the present study, we validated that the SLEscore was associated with eight kinds of immune checkpoints in breast cancer patients, indicating five prognostic genes may act as potential therapeutic targets.

Increasing studies have confirmed that breast cancer evolves on account of a close interaction with TME (45). The ESTIMATE algorithm substantiated that the high expression levels of five key genes significantly decreased the overall immune and stromal activity in the TME of breast cancer. These molecules may provoke immune tolerance by changing TME cell infiltration characterizations and evade attack from the immune system by restructuring the TME structures. We also discovered a remarkably negative correlation between expression levels of the key molecules and several kinds of TME infiltration, such as dendritic cell, eosinophil, mast cell, and CD4+ T cell. These cells play an initial tumor-suppressor role, and the present results of TME cell infiltration were in line with the results of survival analyses for these key molecules. Given the inherent inflammation between the two serious disorders, we hypothesized that PANoptosis, an inflammatory programmed cell death, got involved. PANoptosis were beneficial in anti-cancer effects by stimulating cell death defense mechanisms for the host (46). In the present study, we estimated the correlations between SLE and PANoptosis markers which were proved as prognostic biomarkers in breast cancer. Of interest, we found that the SLEscore was significantly related to the expressions of these gene markers, suggesting the dysfunction of PANoptosis in cases with both conditions.

Several limitations in the present study should be highlighted. First, although various MR methods were performed, potential horizontal pleiotropy could not be entirely eliminated. Fortunately, multiple tests for horizontal pleiotropy and sensitivity generated consistent and reliable results, and no evidence of heterogeneity was discovered, confirming this MR analysis’s findings. Second, we observed an insignificant MR-Egger causal estimate p-value (p=0.23) in the present analysis. Generally, the direction and magnitude of MR estimates were consistent among IVW, weighted median, and MR–Egger methods in the present study. MR–Egger estimates may be less precise, while the MR–Egger intercept could denote the absence of pleiotropy (17). Third, although we demonstrated that SLE was genetically associated with the decreased risk of breast cancer in the East Asian population, underlying mechanisms remain unclear and need to be investigated in further studies. Nonetheless, it is worth noting that, Asian females have a lower risk of breast cancer than whites. Given that Asians might be more vulnerable to SLE (47), the decreased risk of breast cancer in Asian females could be partly explained by our MR results that genetic changes in SLE were significantly associated with the decreased risk of breast cancer in East Asian females.

## Conclusions

Our MR analyses suggested that patients with SLE were less susceptible to the risk of breast cancer among East Asians. The present study also provided a roadmap for the stratification of patients with both breast cancer and SLE, which was conducive to improving strategies for individualized follow-up and personalized decision-making.

## Data availability statement

Data analyzed by the present study can be gained from MR-Base (http://www.mrbase.org/), GEO (http://www.ncbi.nlm.nih.gov/geo/) (Under accession: GSE65391 and GSE42568), and TCGA (https://portal.gdc.cancer.gov/) databases (Under accession: TCGA-BRCA).

## Author contributions

WL and RW concepted the project. WL, RW, and WW acquired the data and performed analyses. WL and RW drafted the manuscript, with critical feedback from WW. All authors contributed to the article and approved the submitted version.

## Supplementary material

Supplementary Figure 1 Study flowchart of the present MR analysis. The MR method follows three assumptions: 1. the IVs are robustly associated with SLE; 2. the IVs affect breast cancer only through the effects on SLE; 3. the IVs are independent of any confounder. MR, Mendelian randomization; IV, instrumental variable; SLE, systemic lupus erythematosus.

Supplementary Figure 2 The correlation between the ESTIMATE score and TTK expression.

Supplementary Figure 3 The correlation between the ESTIMATE score and HMMR expression.

Supplementary Figure 4 The correlation between the ESTIMATE score and RACGAP1 expression.
