# Supplementary figures and images for: Exploring the causality and pathogenesis of systemic lupus erythematosus in breast cancer based on Mendelian randomization and transcriptome data analyses
Source: Front Immunol. 2023 Jan 16;13:1029884. doi: 10.3389/fimmu.2022.1029884 (PMC9885086; doi:10.3389/fimmu.2022.1029884)

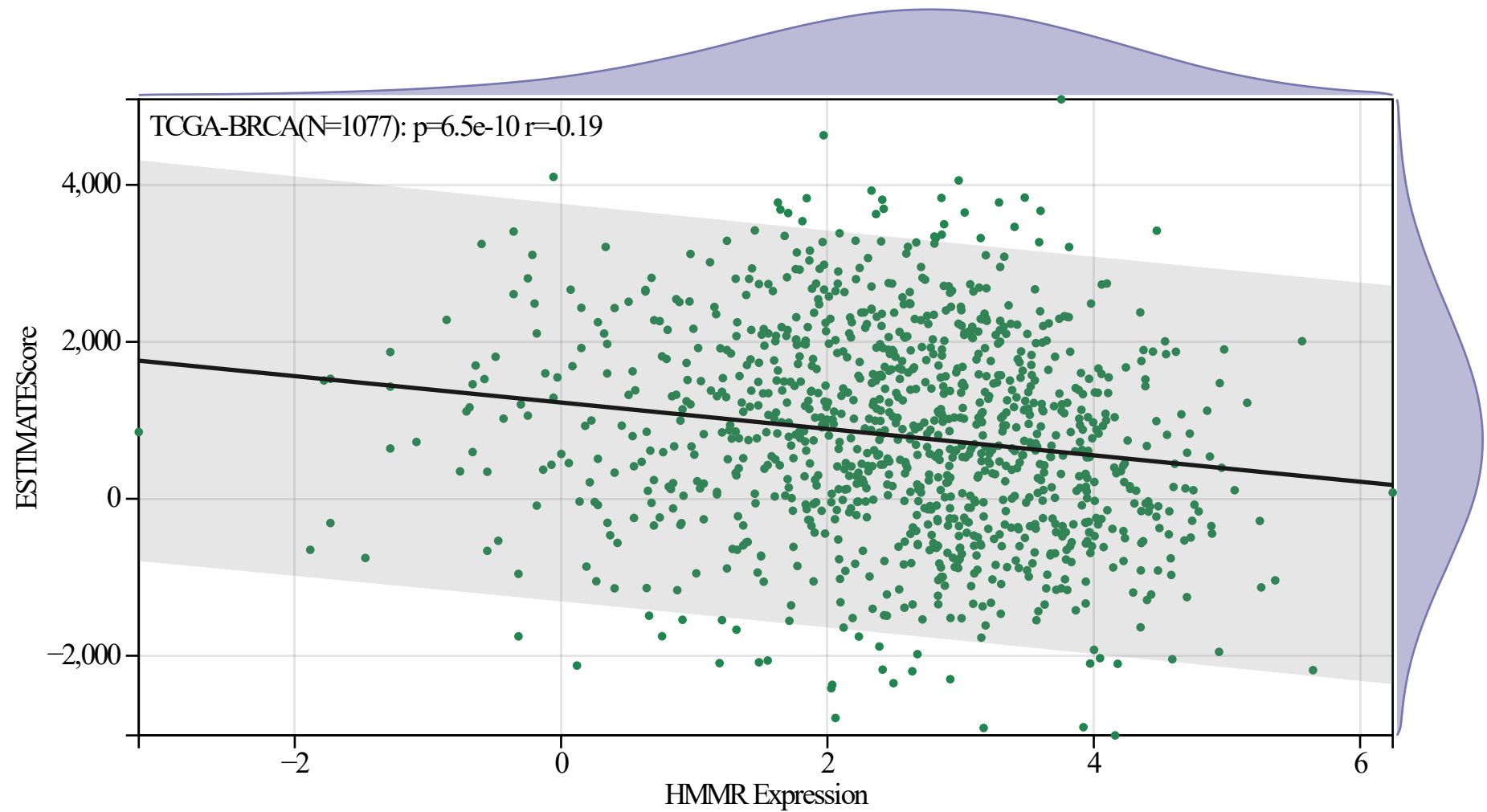

Supplement: Supplementary Figure 1 — Study flowchart of the present MR analysis. The MR method follows three assumptions: 1. the IVs are robustly associated with SLE; 2. the IVs affect breast cancer only through the effects on SLE; 3. the IVs are independent of any confounder. MR, Mendelian randomization; IV, instrumental variable; SLE, systemic lupus erythematosus. [file DataSheet_1.pdf]

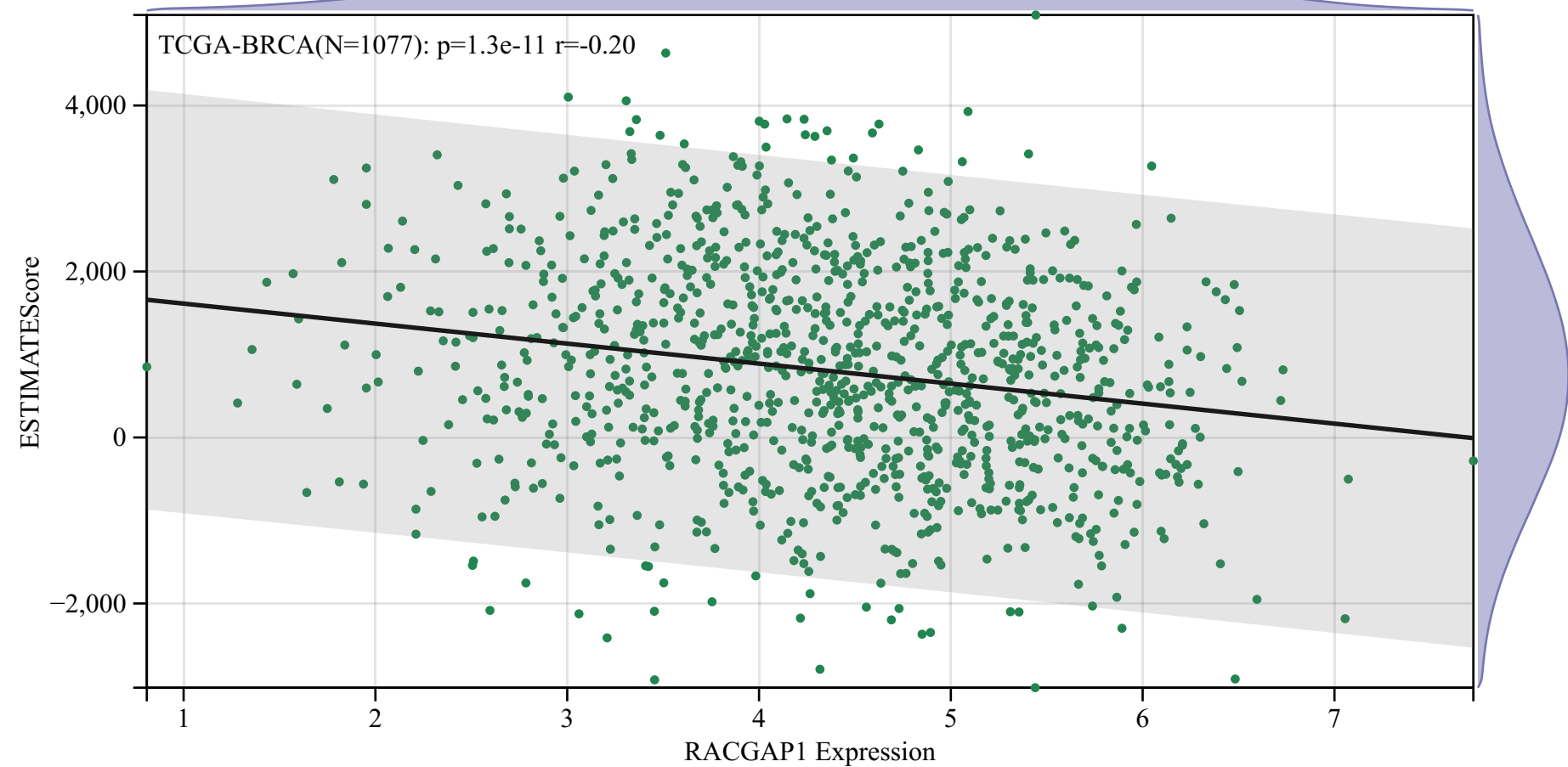

Supplement: Supplementary Figure 2 — The correlation between the ESTIMATE score and TTK expression. [file DataSheet_2.pdf]

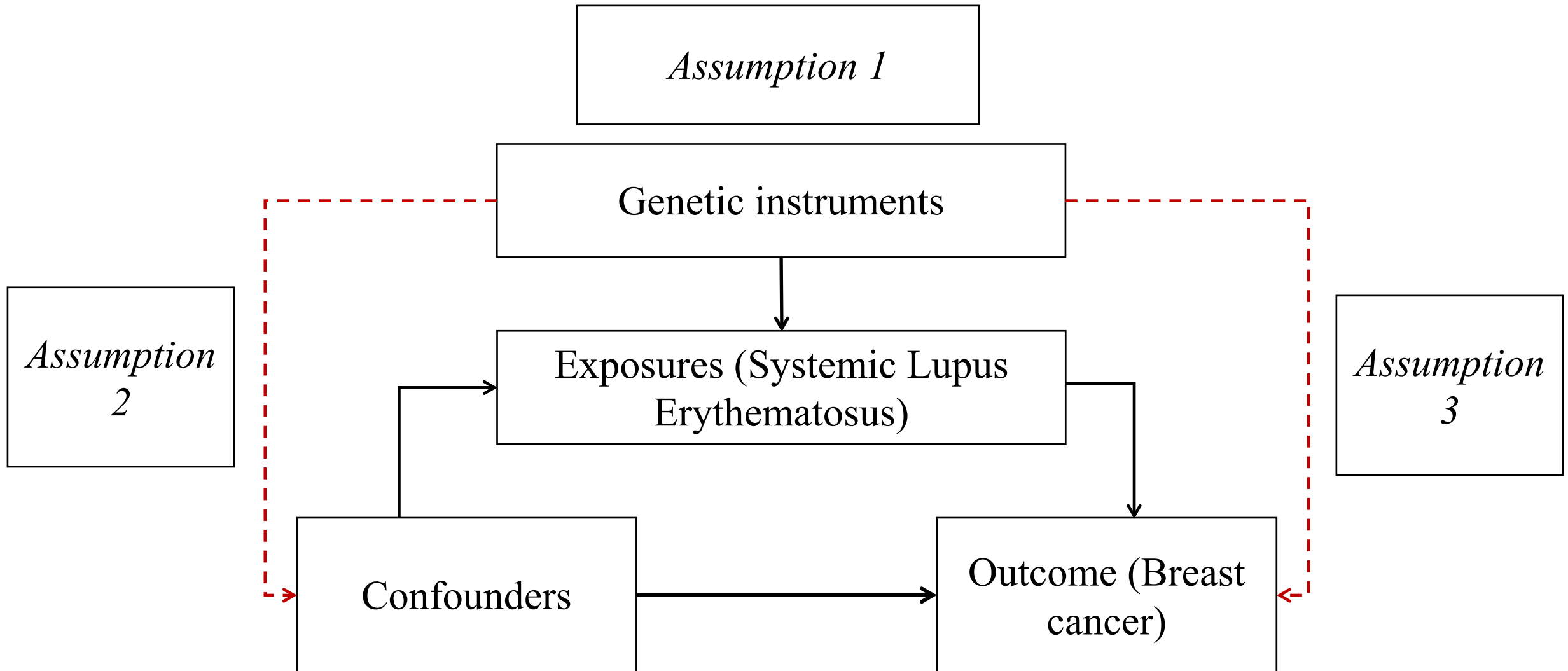

Supplement: Supplementary Figure 3 — The correlation between the ESTIMATE score and HMMR expression. [file DataSheet_3.pdf]

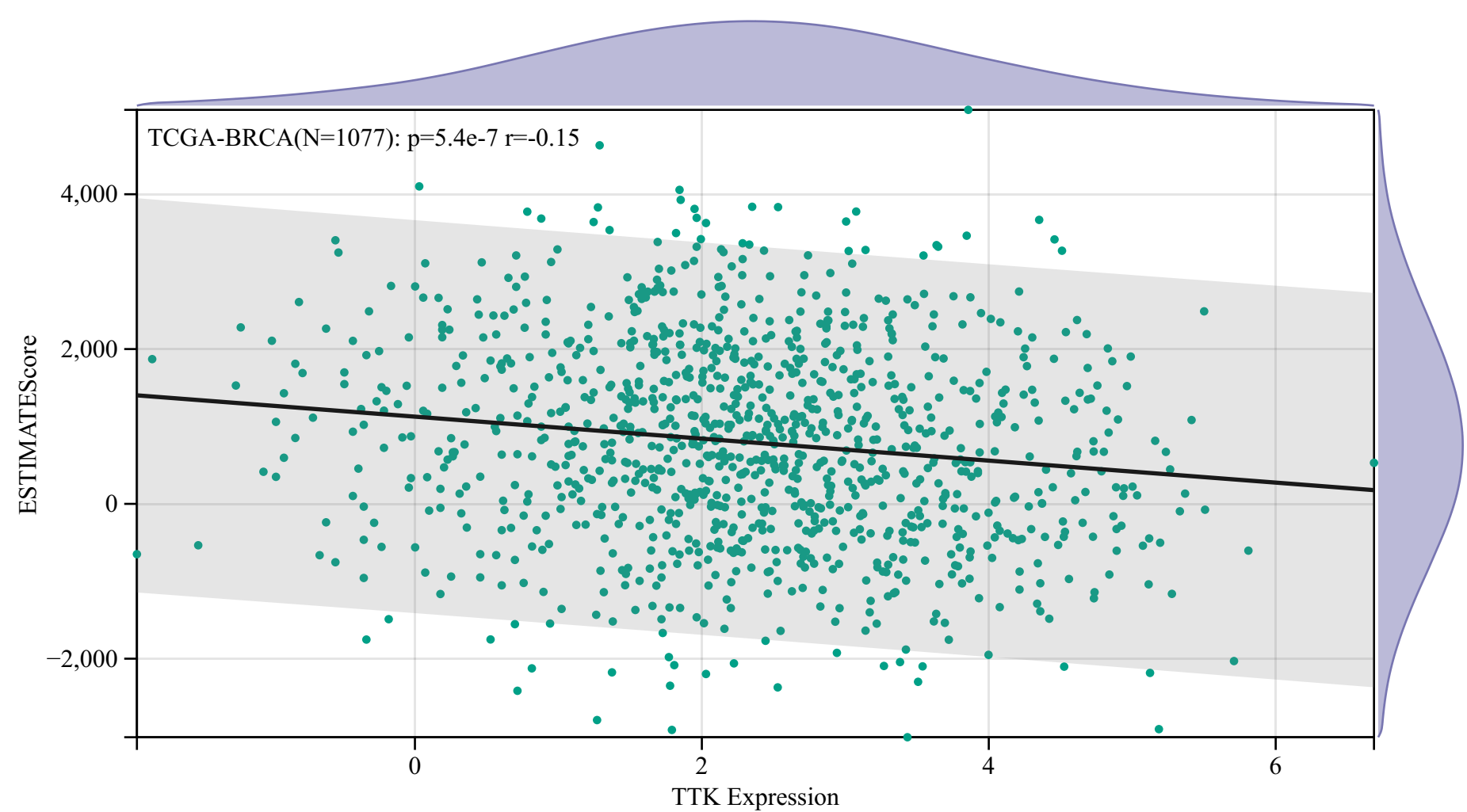

Supplement: Supplementary Figure 4 — The correlation between the ESTIMATE score and RACGAP1 expression. [file DataSheet_4.pdf]
