# Supplementary material for: Exploring the causality and pathogenesis of systemic lupus erythematosus in breast cancer based on Mendelian randomization and transcriptome data analyses
Source: Front Immunol. 2023 Jan 16;13:1029884. doi: 10.3389/fimmu.2022.1029884 (PMC9885086; doi:10.3389/fimmu.2022.1029884)
Supplement: Supplementary file 5 [file DataSheet_5.docx]

| **Supplementary Table S1. Sensitivity analysis.** | | | |  |
| --- | --- | --- | --- | --- |
| Exposure | MR-Egger Test | | |  |
|  | Intercept | SE | p-value |  |
| Breast cancer | 0.0026 | 0.0044 | 0.56 |  |
| ER+ Breast cancer | 0.0023 | 0.005 | 0.65 |  |
| ER- Breast cancer | 0.0023 | 0.005 | 0.65 |  |
| Breast cancer | 0.0047 | 0.015 | 0.754 |  |
| SLE: Systemic Lupus Erythematosus; SE: standard error | | | |  |
|  |  |  |  |  |

| **Supplementary Table S2. MR-PRESSO Tests** | | | | |
| --- | --- | --- | --- | --- |
| Exposure | Ethnicity | MR-PRESSO | | |
|  |  | beta | SE | p value |
| Breast cancer | European | -0.00533307 | 0.00446378 | 0.24 |
| ER+ Breast cancer | European | -0.00635159 | 0.00542750 | 0.25 |
| ER- Breast cancer | European | 0.00809478 | 0.00695122 | 0.25 |
| Breast cancer | East Asian | -0.05083228 | 0.01643043 | **0.004*** |
| *, statistically significant | | | | |

| **Supplementary table S3. Results of WCGNA** | | |
| --- | --- | --- |
| Gene | dynamicModule | mergeModule |
| ABHD11 | turquoise | turquoise |
| ABLIM1 | turquoise | turquoise |
| ACACB | turquoise | turquoise |
| ACKR1 | turquoise | turquoise |
| ACKR3 | turquoise | turquoise |
| ACO1 | turquoise | turquoise |
| ACSL1 | turquoise | turquoise |
| ACSL4 | turquoise | turquoise |
| ACSL5 | turquoise | turquoise |
| ACSM5 | turquoise | turquoise |
| ACTA2 | turquoise | turquoise |
| ADAMTS1 | turquoise | turquoise |
| ADD3 | turquoise | turquoise |
| ADM | turquoise | turquoise |
| ADRB2 | turquoise | turquoise |
| AKR1C1 | turquoise | turquoise |
| AKR1C2 | turquoise | turquoise |
| AKR1C3 | turquoise | turquoise |
| ALDH1A1 | turquoise | turquoise |
| ALDH1A2 | turquoise | turquoise |
| ALPL | turquoise | turquoise |
| ANGPTL4 | turquoise | turquoise |
| ANXA1 | turquoise | turquoise |
| AOC3 | turquoise | turquoise |
| ARID5B | turquoise | turquoise |
| ARL4A | turquoise | turquoise |
| ATF3 | turquoise | turquoise |
| ATP6AP1 | turquoise | turquoise |
| AVPR2 | turquoise | turquoise |
| BCAS4 | turquoise | turquoise |
| BIN1 | turquoise | turquoise |
| BMP6 | turquoise | turquoise |
| BMX | turquoise | turquoise |
| C2CD2 | turquoise | turquoise |
| CA4 | turquoise | turquoise |
| CACFD1 | turquoise | turquoise |
| CANT1 | turquoise | turquoise |
| CAT | turquoise | turquoise |
| CAV1 | turquoise | turquoise |
| CCDC69 | turquoise | turquoise |
| CCL2 | turquoise | turquoise |
| CCND2 | turquoise | turquoise |
| CD248 | turquoise | turquoise |
| CDKN1C | turquoise | turquoise |
| CELF2 | turquoise | turquoise |
| CFI | turquoise | turquoise |
| CLDN5 | turquoise | turquoise |
| CLIC2 | turquoise | turquoise |
| CLIP4 | turquoise | turquoise |
| COL11A1 | turquoise | turquoise |
| COL17A1 | turquoise | turquoise |
| CORO2A | turquoise | turquoise |
| COX6C | turquoise | turquoise |
| CPED1 | turquoise | turquoise |
| CPM | turquoise | turquoise |
| CSRP1 | turquoise | turquoise |
| CTSG | turquoise | turquoise |
| DCTPP1 | turquoise | turquoise |
| DHRS3 | turquoise | turquoise |
| DMTN | turquoise | turquoise |
| DPP4 | turquoise | turquoise |
| DPYSL2 | turquoise | turquoise |
| DUSP1 | turquoise | turquoise |
| EHBP1 | turquoise | turquoise |
| EMP1 | turquoise | turquoise |
| ENPP2 | turquoise | turquoise |
| EPHB1 | turquoise | turquoise |
| ESRP1 | turquoise | turquoise |
| ETS2 | turquoise | turquoise |
| EZR | turquoise | turquoise |
| F13A1 | turquoise | turquoise |
| FABP4 | turquoise | turquoise |
| FAM102A | turquoise | turquoise |
| FAM13A | turquoise | turquoise |
| FAM171A1 | turquoise | turquoise |
| FAP | turquoise | turquoise |
| FBLN1 | turquoise | turquoise |
| FBLN2 | turquoise | turquoise |
| FBLN5 | turquoise | turquoise |
| FGL2 | turquoise | turquoise |
| FKBP4 | turquoise | turquoise |
| FMO2 | turquoise | turquoise |
| FOS | turquoise | turquoise |
| FOXC1 | turquoise | turquoise |
| FOXN3 | turquoise | turquoise |
| FOXO1 | turquoise | turquoise |
| FSTL1 | turquoise | turquoise |
| FXYD1 | turquoise | turquoise |
| FZD7 | turquoise | turquoise |
| GABARAPL1 | turquoise | turquoise |
| GBE1 | turquoise | turquoise |
| GIMAP6 | turquoise | turquoise |
| GLYAT | turquoise | turquoise |
| GNG11 | turquoise | turquoise |
| GPM6B | turquoise | turquoise |
| GSN | turquoise | turquoise |
| GYG2 | turquoise | turquoise |
| GYPC | turquoise | turquoise |
| HADH | turquoise | turquoise |
| HBA2 | turquoise | turquoise |
| HBB | turquoise | turquoise |
| HOXC13 | turquoise | turquoise |
| HPSE2 | turquoise | turquoise |
| HSD17B11 | turquoise | turquoise |
| ICAM2 | turquoise | turquoise |
| IFNGR1 | turquoise | turquoise |
| IRF7 | turquoise | turquoise |
| ITGA6 | turquoise | turquoise |
| ITGA7 | turquoise | turquoise |
| ITIH5 | turquoise | turquoise |
| ITM2A | turquoise | turquoise |
| JPH2 | turquoise | turquoise |
| KANK1 | turquoise | turquoise |
| KAT2B | turquoise | turquoise |
| KCNJ8 | turquoise | turquoise |
| KCTD12 | turquoise | turquoise |
| KIF22 | turquoise | turquoise |
| KLF10 | turquoise | turquoise |
| KLF2 | turquoise | turquoise |
| KLF4 | turquoise | turquoise |
| KLF6 | turquoise | turquoise |
| KLHL29 | turquoise | turquoise |
| KRT8 | turquoise | turquoise |
| LAMTOR2 | turquoise | turquoise |
| LDHB | turquoise | turquoise |
| LEP | turquoise | turquoise |
| LEPR | turquoise | turquoise |
| LIMS2 | turquoise | turquoise |
| LLGL2 | turquoise | turquoise |
| LMO2 | turquoise | turquoise |
| LPL | turquoise | turquoise |
| LRP1 | turquoise | turquoise |
| LRRN3 | turquoise | turquoise |
| LTBP4 | turquoise | turquoise |
| MARCKSL1 | turquoise | turquoise |
| MB | turquoise | turquoise |
| MBOAT2 | turquoise | turquoise |
| MBOAT7 | turquoise | turquoise |
| MCAM | turquoise | turquoise |
| MDFIC | turquoise | turquoise |
| MET | turquoise | turquoise |
| METTL7A | turquoise | turquoise |
| MFGE8 | turquoise | turquoise |
| MGLL | turquoise | turquoise |
| MIA | turquoise | turquoise |
| MIF | turquoise | turquoise |
| MISP | turquoise | turquoise |
| MMD | turquoise | turquoise |
| MME | turquoise | turquoise |
| MMP11 | turquoise | turquoise |
| MMP28 | turquoise | turquoise |
| MOCS1 | turquoise | turquoise |
| MRAS | turquoise | turquoise |
| MT1E | turquoise | turquoise |
| MT1X | turquoise | turquoise |
| MXRA7 | turquoise | turquoise |
| MYCBP2 | turquoise | turquoise |
| MYCT1 | turquoise | turquoise |
| MYL9 | turquoise | turquoise |
| MYLK | turquoise | turquoise |
| NDN | turquoise | turquoise |
| NDRG2 | turquoise | turquoise |
| NEDD9 | turquoise | turquoise |
| NFIB | turquoise | turquoise |
| NFIL3 | turquoise | turquoise |
| NIPSNAP1 | turquoise | turquoise |
| NIPSNAP3B | turquoise | turquoise |
| NME3 | turquoise | turquoise |
| NMT2 | turquoise | turquoise |
| NOTCH4 | turquoise | turquoise |
| NPR1 | turquoise | turquoise |
| NPR2 | turquoise | turquoise |
| NPY2R | turquoise | turquoise |
| NR3C1 | turquoise | turquoise |
| NRN1 | turquoise | turquoise |
| NT5E | turquoise | turquoise |
| NUP210 | turquoise | turquoise |
| NVL | turquoise | turquoise |
| P3H4 | turquoise | turquoise |
| PAFAH1B3 | turquoise | turquoise |
| PAGR1 | turquoise | turquoise |
| PARM1 | turquoise | turquoise |
| PC | turquoise | turquoise |
| PDGFRA | turquoise | turquoise |
| PDZD2 | turquoise | turquoise |
| PECAM1 | turquoise | turquoise |
| PELI1 | turquoise | turquoise |
| PFKFB3 | turquoise | turquoise |
| PHLDB1 | turquoise | turquoise |
| PID1 | turquoise | turquoise |
| PLAGL1 | turquoise | turquoise |
| PLIN2 | turquoise | turquoise |
| PLS3 | turquoise | turquoise |
| PLSCR4 | turquoise | turquoise |
| PLTP | turquoise | turquoise |
| PNPLA2 | turquoise | turquoise |
| POLR3K | turquoise | turquoise |
| PPP1R12B | turquoise | turquoise |
| PPP1R15A | turquoise | turquoise |
| PPP1R16B | turquoise | turquoise |
| PRNP | turquoise | turquoise |
| PRR15L | turquoise | turquoise |
| PTGDS | turquoise | turquoise |
| PTGS2 | turquoise | turquoise |
| PTK6 | turquoise | turquoise |
| PTPRM | turquoise | turquoise |
| QKI | turquoise | turquoise |
| RASSF7 | turquoise | turquoise |
| RCAN1 | turquoise | turquoise |
| RCBTB2 | turquoise | turquoise |
| RECK | turquoise | turquoise |
| RGCC | turquoise | turquoise |
| RGL1 | turquoise | turquoise |
| RGS2 | turquoise | turquoise |
| RHOQ | turquoise | turquoise |
| RNASE1 | turquoise | turquoise |
| S100A14 | turquoise | turquoise |
| S100A4 | turquoise | turquoise |
| S1PR1 | turquoise | turquoise |
| SAMD4A | turquoise | turquoise |
| SDC1 | turquoise | turquoise |
| SELP | turquoise | turquoise |
| SERPINF1 | turquoise | turquoise |
| SERPING1 | turquoise | turquoise |
| SGCE | turquoise | turquoise |
| SGK1 | turquoise | turquoise |
| SLC16A6 | turquoise | turquoise |
| SLC16A7 | turquoise | turquoise |
| SLC27A6 | turquoise | turquoise |
| SLC50A1 | turquoise | turquoise |
| SLC7A10 | turquoise | turquoise |
| SLC9A3R1 | turquoise | turquoise |
| SNRNP25 | turquoise | turquoise |
| SOCS3 | turquoise | turquoise |
| SORD | turquoise | turquoise |
| SOX12 | turquoise | turquoise |
| SPRY1 | turquoise | turquoise |
| SPRY2 | turquoise | turquoise |
| SPTBN1 | turquoise | turquoise |
| SPX | turquoise | turquoise |
| STX11 | turquoise | turquoise |
| STXBP2 | turquoise | turquoise |
| SYN2 | turquoise | turquoise |
| TBC1D4 | turquoise | turquoise |
| TFPI | turquoise | turquoise |
| TGFBR2 | turquoise | turquoise |
| THOC6 | turquoise | turquoise |
| TLE4 | turquoise | turquoise |
| TMOD1 | turquoise | turquoise |
| TNS1 | turquoise | turquoise |
| TP63 | turquoise | turquoise |
| TPD52 | turquoise | turquoise |
| TPM2 | turquoise | turquoise |
| TSHZ2 | turquoise | turquoise |
| TSPAN13 | turquoise | turquoise |
| TSPAN7 | turquoise | turquoise |
| TUBB6 | turquoise | turquoise |
| UGP2 | turquoise | turquoise |
| UNC5B | turquoise | turquoise |
| VWF | turquoise | turquoise |
| YBX3 | turquoise | turquoise |
| ZFP36 | turquoise | turquoise |
| ZNF552 | turquoise | turquoise |
| AKR7A3 | grey | grey |
| ALDOC | grey | grey |
| ANPEP | grey | grey |
| ANXA3 | grey | grey |
| ASS1 | grey | grey |
| CAPG | grey | grey |
| CD24 | grey | grey |
| CEACAM6 | grey | grey |
| CERS2 | grey | grey |
| CFB | grey | grey |
| COL1A1 | grey | grey |
| CSN1S1 | grey | grey |
| CTNNAL1 | grey | grey |
| CTSD | grey | grey |
| CXCR4 | grey | grey |
| DSC3 | grey | grey |
| ECHDC3 | grey | grey |
| ENO2 | grey | grey |
| EVL | grey | grey |
| FGFR3 | grey | grey |
| GATA3 | grey | grey |
| GOLM1 | grey | grey |
| GSPT2 | grey | grey |
| GSTP1 | grey | grey |
| KRT6B | grey | grey |
| LAMC2 | grey | grey |
| LRRC15 | grey | grey |
| LTB | grey | grey |
| LTF | grey | grey |
| MAGED2 | grey | grey |
| MDK | grey | grey |
| ME1 | grey | grey |
| MUC1 | grey | grey |
| MXI1 | grey | grey |
| MYBPC1 | grey | grey |
| MYC | grey | grey |
| NAT1 | grey | grey |
| NAV2 | grey | grey |
| NCALD | grey | grey |
| NELL2 | grey | grey |
| OAS1 | grey | grey |
| OAS2 | grey | grey |
| OGFRL1 | grey | grey |
| OLFM4 | grey | grey |
| OXCT1 | grey | grey |
| P3H2 | grey | grey |
| PLEK2 | grey | grey |
| PYCARD | grey | grey |
| PYGL | grey | grey |
| RAB31 | grey | grey |
| RET | grey | grey |
| RRAGD | grey | grey |
| S100P | grey | grey |
| SIAH2 | grey | grey |
| SLC12A8 | grey | grey |
| SLC34A2 | grey | grey |
| SLC44A4 | grey | grey |
| SLPI | grey | grey |
| SYCP2 | grey | grey |
| TSPAN1 | grey | grey |
| TUFT1 | grey | grey |
| ABCA5 | blue | blue |
| ABLIM3 | blue | blue |
| AHNAK | blue | blue |
| AK5 | blue | blue |
| AMIGO2 | blue | blue |
| ARHGEF6 | blue | blue |
| ASPM | blue | blue |
| ATP6V0B | blue | blue |
| AURKA | blue | blue |
| BCL6 | blue | blue |
| BIRC5 | blue | blue |
| BOP1 | blue | blue |
| C1orf115 | blue | blue |
| CA12 | blue | blue |
| CAB39L | blue | blue |
| CBX7 | blue | blue |
| CCNA2 | blue | blue |
| CCNB2 | blue | blue |
| CCT3 | blue | blue |
| CDC20 | blue | blue |
| CENPF | blue | blue |
| CENPM | blue | blue |
| COBLL1 | blue | blue |
| CRIM1 | blue | blue |
| CRY2 | blue | blue |
| CTPS1 | blue | blue |
| CXCL10 | blue | blue |
| CYBRD1 | blue | blue |
| DBNDD1 | blue | blue |
| DDX39A | blue | blue |
| DLGAP5 | blue | blue |
| DPP3 | blue | blue |
| DTYMK | blue | blue |
| DUSP6 | blue | blue |
| ECT2 | blue | blue |
| ERCC6L | blue | blue |
| EXOSC4 | blue | blue |
| EZH2 | blue | blue |
| FAXDC2 | blue | blue |
| FBXL6 | blue | blue |
| FCER1A | blue | blue |
| FLAD1 | blue | blue |
| GGCT | blue | blue |
| GSTM2 | blue | blue |
| GTSE1 | blue | blue |
| HGH1 | blue | blue |
| HMGA1 | blue | blue |
| HMMR | blue | blue |
| IFI6 | blue | blue |
| INHBB | blue | blue |
| IRS1 | blue | blue |
| IRS2 | blue | blue |
| ISG15 | blue | blue |
| ITPR1 | blue | blue |
| JADE1 | blue | blue |
| KIF15 | blue | blue |
| KIF23 | blue | blue |
| LAGE3 | blue | blue |
| LIG1 | blue | blue |
| LMNB1 | blue | blue |
| LPCAT1 | blue | blue |
| LSM4 | blue | blue |
| LSR | blue | blue |
| MBNL2 | blue | blue |
| MCM4 | blue | blue |
| MELK | blue | blue |
| MMP9 | blue | blue |
| MRPL12 | blue | blue |
| NCAPG | blue | blue |
| NDC80 | blue | blue |
| NME1 | blue | blue |
| NR3C2 | blue | blue |
| NUSAP1 | blue | blue |
| OASL | blue | blue |
| OSBPL1A | blue | blue |
| PARP1 | blue | blue |
| PCNA | blue | blue |
| PDGFD | blue | blue |
| PELI2 | blue | blue |
| PER2 | blue | blue |
| PIK3R1 | blue | blue |
| PLAUR | blue | blue |
| PPL | blue | blue |
| PRC1 | blue | blue |
| PTTG1 | blue | blue |
| RACGAP1 | blue | blue |
| RAD51AP1 | blue | blue |
| RBPMS | blue | blue |
| RCC1 | blue | blue |
| RPL39L | blue | blue |
| RUSC1 | blue | blue |
| SDF2L1 | blue | blue |
| SHCBP1 | blue | blue |
| SLC52A2 | blue | blue |
| SLC7A5 | blue | blue |
| SMC4 | blue | blue |
| SNRPB | blue | blue |
| SPC25 | blue | blue |
| SQLE | blue | blue |
| ST14 | blue | blue |
| STEAP4 | blue | blue |
| STMN1 | blue | blue |
| TACC3 | blue | blue |
| TAP1 | blue | blue |
| TESC | blue | blue |
| TGFBR3 | blue | blue |
| TIMELESS | blue | blue |
| TIMM17B | blue | blue |
| TIPARP | blue | blue |
| TMEM97 | blue | blue |
| TOP2A | blue | blue |
| TRIP13 | blue | blue |
| TROAP | blue | blue |
| TTC28 | blue | blue |
| TTK | blue | blue |
| TTLL12 | blue | blue |
| TUBA1C | blue | blue |
| TXNIP | blue | blue |
| TYMP | blue | blue |
| TYMS | blue | blue |
| UBE2C | blue | blue |
| UBE2S | blue | blue |
| WLS | blue | blue |
| WGCNA: Weighted Gene Co-Expression Network Analysis | | |
