# Supplementary material for: Exploring the causality and pathogenesis of systemic lupus erythematosus in breast cancer based on Mendelian randomization and transcriptome data analyses
Source: Front Immunol. 2023 Jan 16;13:1029884. doi: 10.3389/fimmu.2022.1029884 (PMC9885086; doi:10.3389/fimmu.2022.1029884)
Supplement: Supplementary file 6 [file DataSheet_6.docx]

**MR checklist of recommended items to address in reports of Mendelian randomization studies**

| **Item No.** | **Section** | **Checklist item** | **Section (paragraph number)** |
| --- | --- | --- | --- |
| 1 | **TITLE and ABSTRACT** | Indicate Mendelian randomization as the study’s design in the title and/or the abstract if that is a main purpose of the study | *Title page* |
|  | **INTRODUCTION** |  |  |
| 2 | **Background** | Explain the scientific background and rationale for the reported study. What is the exposure? Is a potential causal relationship between exposure and outcome plausible? Justify why MR is a helpful method to address the study question | *Introduction (paragraphs 1-2)* |
| 3 | **Objectives** | State specific objectives clearly, including pre-specified causal hypotheses (if any). State that MR is a method that, under specific assumptions, intends to estimate causal effects | *Introduction (paragraph 2), Methods (Generation of Genetic Instruments)* |
|  | **METHODS** |  |  |
| 4 | **Study design and data sources** | Present key elements of the study design early in the article. Consider including a table listing sources of data for all phases of the study. For each data source contributing to the analysis, describe the following: | *Methods (Data Sources, Generation of Genetic Instruments), Table 1* |
|  | a) | Setting: Describe the study design and the underlying population, if possible. Describe the setting, locations, and relevant dates, including periods of recruitment, exposure, follow-up, and data collection, when available. | *Methods (Data Sources), Table 1* |
|  | b) | Participants: Give the eligibility criteria, and the sources and methods of selection of participants. Report the sample size, and whether any power or sample size calculations were carried out prior to the main analysis | *Methods (Data Sources), Table 1* |
|  | c) | Describe measurement, quality control and selection of genetic variants | *Methods (Generation of Genetic Instruments)* |
|  | d) | For each exposure, outcome, and other relevant variables, describe methods of assessment and diagnostic criteria for diseases | *Methods (Data Sources)* |
|  | e) | Provide details of ethics committee approval and participant informed consent, if relevant | *Not applicable* |
| 5 | **Assumptions** | Explicitly state the three core IV assumptions for the main analysis (relevance, independence and exclusion restriction) as well assumptions for any additional or sensitivity analysis | *Methods (Generation of Genetic Instruments)* |
| 6 | **Statistical methods: main analysis** | Describe statistical methods and statistics used | *Methods (Generation of Genetic Instruments, Two-Sample and Multivariate MR Analysis), Table 2* |
|  | a) | Describe how quantitative variables were handled in the analyses (i.e., scale, units, model) | *Table 2* |
|  | b) | Describe how genetic variants were handled in the analyses and, if applicable, how their weights were selected | *Methods (Generation of Genetic Instruments, Two-Sample and Multivariate MR Analysis)* |
|  | c) | Describe the MR estimator (e.g. two-stage least squares, Wald ratio) and related statistics. Detail the included covariates and, in case of two-sample MR, whether the same covariate set was used for adjustment in the two samples | *Methods (Two-Sample and Multivariate MR Analysis)* |
|  | d) | Explain how missing data were addressed | *Not applicable* |
|  | e) | If applicable, indicate how multiple testing was addressed | *Methods (Two-Sample and Multivariate MR Analysis)* |
| 7 | **Assessment of assumptions** | Describe any methods or prior knowledge used to assess the assumptions or justify their validity | *Methods (Removal of Horizontal Pleiotropy and Sensitivity Analyses)* |
| 8 | **Sensitivity analyses and additional analyses** | Describe any sensitivity analyses or additional analyses performed (e.g. comparison of effect estimates from different approaches, independent replication, bias analytic techniques, validation of instruments, simulations) | *Methods (Removal of Horizontal Pleiotropy and Sensitivity Analyses)* |
| 9 | **Software and pre-registration** |  |  |
|  | a) | Name statistical software and package(s), including version and settings used | *Methods (Statistical Analysis)* |
|  | b) | State whether the study protocol and details were pre-registered (as well as when and where) | *Not applicable* |
|  | **RESULTS** |  |  |
| 10 | **Descriptive data** |  |  |
|  | a) | Report the numbers of individuals at each stage of included studies and reasons for exclusion. Consider use of a flow-diagram | *Results (Selection of SNPs)* |
|  | b) | Report summary statistics for phenotypic exposure(s), outcome(s) and other relevant variables (e.g. means, SDs, proportions) | *Not applicable* |
|  | c) | If the data sources include meta-analyses of previous studies, provide the assessments of heterogeneity across these studies | *Not applicable* |
|  | d) | For *two-sample* Mendelian randomisation:  i.  Provide justification of the similarity of the genetic variant-exposure associations between the exposure and outcome samples  ii.  Provide information on the number of individuals who were in both samples for the exposure and for the outcome | *Not applicable* |
| 11 | **Main results** |  |  |
|  | a) | Report the associations between genetic variant and exposure, and between genetic variant and outcome, preferably on an interpretable scale | *Results (Genetic Susceptibility to SLE and BRCA Risk)* |
|  | b) | Report MR estimates of the relationship between exposure and outcome, and the measures of uncertainty from the MR analysis, on an interpretable scale, such as odds ratio or relative risk per SD difference | *Results (Genetic Susceptibility to SLE and BRCA Risk)* |
|  | c) | If relevant, consider translating estimates of relative risk into absolute risk for a meaningful time period | *Not applicable* |
|  | d) | Consider plots to visualize results (e.g. forest plot, scatterplot of associations between genetic variants and outcome versus between genetic variants and exposure) | *Figures 2, Table 2* |
| 12 | **Assessment of assumptions** |  |  |
|  | a) | Report the assessment of the validity of the assumptions | *Results (Sensitivity Analyses for MR Estimates), Table S1-2* |
|  | b) | Report any additional statistics (e.g., assessments of heterogeneity across genetic variants, such as I^2^, Q statistic or E-value) | *Results (Sensitivity Analyses for MR Estimates), Table S1-2* |
| 13 | **Sensitivity analyses and additional analyses** |  |  |
|  | a) | Report any sensitivity analyses to assess the robustness of the main results to violations of the assumptions | *Results (Sensitivity Analyses for MR Estimates), Table S1-2* |
|  | b) | Report results from other sensitivity analyses or additional analyses | *Results (Sensitivity Analyses for MR Estimates), Table S1-2* |
|  | c) | Report any assessment of direction of causal relationship (e.g., bidirectional MR) | *Not applicable* |
|  | d) | When relevant, report and compare with estimates from non-MR analyses | *Not applicable* |
|  | e) | Consider additional plots to visualize results (e.g., leave-one-out analyses) | *Figures 2* |
|  | **DISCUSSION** |  |  |
| 14 | **Key results** | Summarize key results with reference to study objectives | *Discussion (paragraph 1)* |
| 15 | **Limitations** | Discuss limitations of the study, taking into account the validity of the IV assumptions, other sources of potential bias, and imprecision. Discuss both direction and magnitude of any potential bias and any efforts to address them | *Discussion (paragraph 7)* |
| 16 | **Interpretation** |  |  |
|  | a) | Meaning: Give a cautious overall interpretation of results in the context of their limitations and in comparison with other studies | *Discussion (paragraph 2-3)* |
|  | b) | Mechanism: Discuss underlying biological mechanisms that could drive a potential causal relationship between the investigated exposure and the outcome, and whether the gene-environment equivalence assumption is reasonable. Use causal language carefully, clarifying that IV estimates may provide causal effects only under certain assumptions | *Discussion (paragraphs 4-6)* |
|  | c) | Clinical relevance: Discuss whether the results have clinical or public policy relevance, and to what extent they inform effect sizes of possible interventions | *Discussion (paragraph 1-3)* |
| 17 | **Generalizability** | Discuss the generalizability of the study results (a) to other populations, (b) across other exposure periods/timings, and (c) across other levels of exposure | *Not applicable* |
|  | **OTHER INFORMATION** |  |  |
| 18 | **Funding** | Describe sources of funding and the role of funders in the present study and, if applicable, sources of funding for the databases and original study or studies on which the present study is based | *Section (Funding)* |
| 19 | **Data and data sharing** | Provide the data used to perform all analyses or report where and how the data can be accessed, and reference these sources in the article. Provide the statistical code needed to reproduce the results in the article, or report whether the code is publicly accessible and if so, where | *Section (Availability of data)* |
| 20 | **Conflicts of Interest** | All authors should declare all potential conflicts of interest | *Section (Competing interests)* |
